# The Fibrosis and Immunological Features of Hypochlorous Acid Induced Mouse Model of Systemic Sclerosis

**DOI:** 10.3389/fimmu.2019.01861

**Published:** 2019-08-20

**Authors:** Meng Meng, Jieqiong Tan, Weilin Chen, Qian Du, Bin Xie, Nian Wang, Honglin Zhu, Kangkai Wang

**Affiliations:** ^1^Department of Pathology, Xiangya Hospital, Central South University, Changsha, China; ^2^Department of Pathophysiology, School of Basic Medical Science, Central South University, Changsha, China; ^3^The Center for Medical Genetics, School of Life Science, Central South University, Changsha, China; ^4^Department of Rheumatology, Xiangya Hospital, Central South University, Changsha, China; ^5^Key Laboratory of Sepsis Translational Medicine of Hunan, Central South University, Changsha, China; ^6^Department of Laboratory Animals, Xiangya School of Medicine, Central South University, Changsha, China

**Keywords:** HOCl-induced mice, immune cell infiltration, pro-inflammatory mediators, fibrosis, SSc

## Abstract

Fibrotic animal models are critical for the pathogenesis investigations and drug explorations in systemic sclerosis (SSc). The bleomycin (BLM)-induced mouse model is the classical and most widely used fibrosis model. However, traditional subcutaneous injection of BLM rarely induced diffuse skin and lung lesions. Hypochlorous acid (HOCl)-induced mice are a more representative model that have diffuse cutaneous lesions, lung fibrosis and renal involvement. However, the fibrotic and immunological features of this model are not fully elucidated. Here, we injected BALB/c mice subcutaneously with HOCl used at different concentrations of HOCl (1:55, 1:70, and 1:110 NaClO: KH2PO4, hereafter named HOCl55, HOCl70, and HOCl110, respectively) for 6 weeks to induce fibrosis, and also used HOCl110 at different time course (4, 5, and 6 weeks). Morphological changes were observed via HE and Masson's trichrome staining. Immunohistochemistry or real-time PCR was used to detect inflammatory infiltrates, important fibrosis pathways and pro-inflammatory mediator expression. Flow cytometry was used to detect the alteration of immune cells in mouse spleen. Skin and lung fibrosis were most obvious in the HOCl55 group compared to lower concentration groups. In the HOCl110 group, dominant inflammatory infiltrates were found after 5 weeks, and significant fibrosis was found after 6 weeks. Then we explored the fibrosis and immunological profiles in the HOCl110 (6 weeks) group. Important fibrosis pathway proteins such as TGF-β, NF-κB, Smad3, p-Smad3, STAT3, and p-STAT3 were significantly elevated at week 6 in the HOCl110 group. Increased infiltration of CD4+T cells, CD8+T cells, CD20+B cells, and myofibroblasts was found both in skin and lung tissues. However, decreased CD4+T cells, CD8+T cells, monocytes and macrophages and increased CD19+B cells were found in the spleen tissues. The mRNA expression of fibrosis mediators such as IL-1β, IL-6, IL-17, IL-33, TNF-α, and CTGF was also upregulated in skin and lung tissues. In conclusion, HOCl induced fibrosis mouse model displayed systemic immune cell infiltration, pro-inflammatory mediator release, vasculopathy and fibrosis, which better mimicked human SSc than BLM-induced mice.

## Introduction

Systemic sclerosis (SSc) is a highly heterogeneous autoimmune disease. The main clinical manifestations of SSc include skin and multiple organ fibrosis, Raynaud's phenomenon (RP), renal crisis, pulmonary arterial hypertension, gastro-esophageal reflux and digital ulceration (1, 2). Lung fibrosis and pulmonary arterial hypertension contribute to the high mortality (3). The pathogenesis of SSc is very complicated, and both perturbed innate and adaptive immune responses participate in the initiation and development of SSc (4, 5). Immune cells (CD4^+^ T cells, CD8^+^ T cells, and B cells) (6–8), platelets (9), endothelial cells (10), fibroblasts (11) and a large number of autocrine and paracrine factors are involved. These cells release several cytokines, such as interleukin-1 (IL-1) β, IL-6, IL-17, and IL-33. Tumor necrosis factor (TNF)-α, connective tissue growth factor (CTGF) and platelet-derived growth factor receptor alpha (PDGFRA) exert pro-inflammatory and/or pro-fibrotic effects. Meanwhile, many signaling pathways play important roles in SSc fibrosis, such as transforming growth factor beta (TGF-β), toll-like receptor 4 (TLR4) and IL-6/Signal transducers and activators of transcription (STAT3) signaling. TGF-β signaling is commonly viewed as playing a critical role in SSc fibrosis (12, 13). Inhibition of TGF-β signaling protects against bleomycin (BLM)-induced fibrosis in animal models (14, 15). High levels of TLR4 were also found in SSc skin and lung biopsies. TLR4 enhances the sensitivity of fibroblasts to the profibrotic stimulatory effect of TGF-β and serves as the switch for converting self-limited tissue repair into intractable fibrosis. Genetic targeting of TLR4 or its endogenous ligands ameliorates experimental fibrosis in mouse models of SSc (16). STAT3 is a member of the transcription factor family that transduces cellular signals from a number of soluble growth factors and cytokines, such as IL-6 family cytokines, epidermal growth factor (EGF) and platelet-derived growth factor (PDGF). STAT3 can integrate multiple profibrotic signals and is regarded as a key checkpoint in fibroblast activation.

Over the years, many animal models of SSc have been constructed, including exogenous administration of fibrosis-inducing agents and genetic manipulation of fibrosis-related signaling. The former includes BLM, AdTGF-β^223/235^ or reactive oxygen species (ROS)-treated mice. The later includes Tsk-2, TbRIIDk-fib, Fbn-1 mutations, Fli1-KLF5-KO, uPAR-KO, FRA-2 Tg, and Sirt3-KO (17). Each animal model can recapitulate one or more aspects of SSc, and the models play important roles in mechanistic research and preclinical drug development. However, animal models that fully reproduce the pathophysiology of SSc are not available currently. Many drugs have shown excellent anti-fibrosis effects in animal models but have failed in clinical trials (18).

The Hypochlorous acid (HOCl)-induced mouse model of SSc was first constructed by Servettaz et al. This is a more representative model than the BLM-induced model. This model had diffuse cutaneous lesions, lung fibrosis and renal involvement, along with the production of serum anti-DNA topoisomerase 1 autoantibodies. All features seem similar to diffuse cutaneous SSc in humans (19–21). However, no studies have fully characterized the fibrosis and immunological features of HOCl induced mouse model. Here, we first used different concentrations of HOCl, as well as different time courses to induce fibrosis in mice, then we compared the morphological changes in each group. Finally, we studied the alterations of immune cell infiltration in tissues and the expression of important fibrosis mediators and related cytokines in a HOCl-induced fibrosis mouse model.

## Materials and Methods

### Animal Protocol

Six-week-old female BALB/c mice were purchased from Janvier Laboratory (STA, China). All chemical agents were obtained from Macklin Agency (Shanghai, China). Animals received human care in compliance with the guidelines implemented at our institution. The study was performed according to the international, national and institutional rules considering animal experiments, clinical studies and biodiversity rights.

### HOCl-Induced Fibrosis Mouse Model

Mice were randomly distributed into experimental and control groups (*n* = 15). The HOCl-induced mice were induced according to the protocol described by Servettaz et al. (19) with minor modifications. HOCl was produced by adding NaClO solution (active chlorine as 6%) to KH_2_PO_4_ solution (100 mM; pH 6.2) at three different dilutions (1:55, 1:70, and 1:110 NaClO:KH2PO4, hereafter named HOCl55, HOCl70, and HOCl110, respectively). A total of 200 μl of the diluted solution of HOCl was prepared temporaneously and injected intradermally into the shaved backs of the mice, using a 27-gauge needle, every day for 6 weeks (HOCl-injected mice). Control mice received injections of 200 μl of sterilized phosphate buffer saline (PBS-mice).

### BLM-Induced Fibrosis Mouse Model

Subcutaneous injection of BLM could induce dermal fibrosis while rarely causing lung fibrosis. Similarly, tracheal administration of BLM could induce lung fibrosis without skin fibrosis. Therefore, we used both models here. Skin fibrosis was induced by local intracutaneous injections as described previously (22). The mice were challenged with intracutaneous injections of BLM (100 μl of a 100 ug/ml solution in PBS) in defined areas of 1.5 cm^2^ on the upper back every other day for 6 weeks. The control group received intracutaneous injections of 100 μl PBS for 6 weeks. Lung fibrosis was induced by intratracheal instillation of BLM as previously described (23). Mice were randomly divided into experimental and control groups (*n* = 10). When mice were anesthetized, the trachea was separated, 100 μl BLM (3.5 mg/kg, 100 μl saline in the control group) was administered, and then the mice were rapidly rotated for 1 min for BLM distribution in the lungs. After surgery, mice were observed for 6 weeks.

At the end of the sixth week, skin and lung tissues were collected from mice under aseptic conditions and processed for hematoxylin and eosin (HE) staining, Masson's trichrome staining, immunohistochemistry and the detection of mRNA expression of related genes.

### HE and Masson’s Trichrome Staining

The skin or lung tissues were inflated with 10% formalin solution, embedded in paraffin and sectioned. Skin samples were collected near the injection site. Tissue sections were deparaffinated and stained with hematoxylin and eosin for histological examination. For Masson's trichrome staining, sections were treated sequentially with hematoxylin and ferric oxide, acid fuchsin, phosphomolybdic acid, and acetic acid, and then the sections were mounted with neutral gum. Photographs were taken and dermal thickness was measured in the HE sections (Leica, Germany; DMI4000B). We defined the mean distance from the epidermal–dermal junction to the dermal–subcutaneous fat junction and measured 5 different skin sections in every mouse. Two independent observers performed these measurements (23).

### Hydroxyproline Assay

The frozen tissues of the skins and lungs (*n* = 5) were analyzed for hydroxyproline content using a commercially available assay kit using the manufacturer's protocol (MAK008-1KT, Sigma, USA). A standard curve was generated for each assay using a hydroxyproline standard, and the hydroxyproline content in each sample was calculated using this standard curve. Results were expressed as micrograms of hydroxyproline per milligrams of tissue.

### Immunohistochemical Analysis

Skin or lung tissue sections were deparaffinized, and antigen retrieval was performed by incubating the slides with proteinase K (Dako) for 20 min. Slides were then incubated with 3% H_2_O_2_ for 10 min, followed by incubation with 5% bovine serum albumin and 1% rabbit serum to block nonspecific binding. Slides were immunostained with a mouse monoclonal antibody (anti-CD4, anti-CD8, anti-CD19, anti-αSMA, or anti-vimentin) (Sino Biological, China) for 1 h. Other mouse monoclonal antibodies (anti-TGF-β, anti-Smad3, anti-pSmad3, anti-TLR-4, anti-NF-κB, anti-STAT3, or anti-pSTAT3) (Cell Signaling, USA) were incubated for 24 h. The antibody dilution concentrations were 1:100, 1:100, 1:100, 1:50, 1:500, 1:400, and 1:400. After washing in Tris buffered saline-Tween (TBST), slides were incubated with alkaline phosphatase–labeled rabbit anti-mouse secondary antibody (Rockland) for 1 h. Staining was visualized with a diaminobenzidine solution kit (Sigma). Irrelevant isotype-matched antibodies were used as negative controls.

Image-pro Plus was used for immunohistochemical analysis. The mean IOD (intensity optical density) was used to quantify the immunohistochemical expression.

### RNA Isolation and Quantitative Reverse Transcription PCR

As previously described (24), total RNA was isolated from mouse skin or lung tissue using Trizol (Invitrogen Life Technologies) according to the manufacturer's instructions. cDNA was prepared using the Reverse Transcription System (Promega). The expression of related genes was measured using gene-specific primers (shown in Table S1) with SYBR Green (SYBR Premix Ex Taq RT-PCR kit, Takara) and the 7500 real-time PCR system analyzer (Applied Biosystems). GAPDH expression was used as the endogenous control to normalize the sample data. Relative expression levels were calculated using the 2^−ΔΔCt^ method.

### Analysis of Immune Cell Populations in the Spleen

Spleen single-cell suspensions were collected from the mice, filtered with a cell strainer, distributed into 2 sets and were suspended in 50 μL of cold PBS containing 2% fetal calf serum. The first set of cells was stained with V450-conjugated anti-CD45 (BD Bioscience), BB515-conjugated anti-major histocompatibility complex II (MHCII) (BD Bioscience), phycoerythrin-conjugated anti-CD11c (BD Bioscience) for the detection of activated DCs, anti-CD11b^+^/F4/80^+^ for macrophages, and anti-CD11b^+^/Ly6G^+^ for neutrophils. The other set of cells was stained with peridinin chlorophyll protein complex-conjugated anti-CD3 (BD Bioscience), fluorescein isothiocyanate-conjugated anti-CD4 (eBiosience, San Diego, CA, USA), APC-conjugated anti-CD8, and phycoerythrin-conjugated anti-Foxp3 for the detection of T cells. The cells were analyzed by flow cytometry (LSR II; BD Bioscience). Flow cytometry was performed using an BD FACSCantoIIand analyzed by FlowJo software.

### Statistical Analysis

GraphPad Prism software was used for all statistical analysis. Numerical variables with a normal distribution were compared using unpaired *t*-tests. Data with a non-normal distribution were compared using the Mann-Whitney U test. All data are expressed as the mean ± SEM. *P* < 0.05 was considered statistically significant.

## Results

### HOCl-Induced Dermal Inflammation and Fibrosis in a Dose- and Time- Dependent Manner

**Figure 1:**
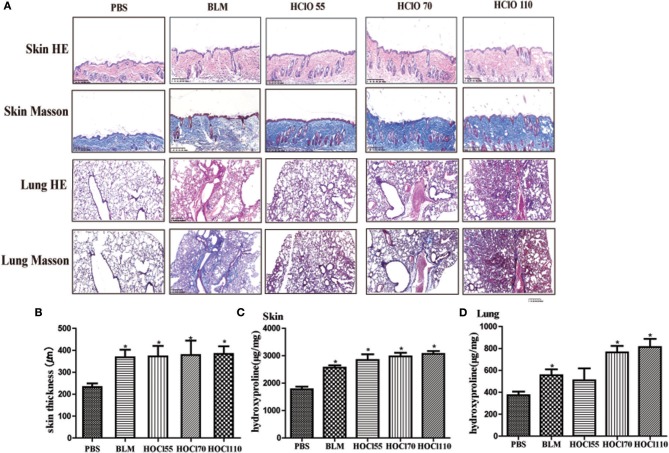
HOCl-induced dermal inflammation and fibrosis in a dose-dependent manner. Different dilution of HOCl (HOCl 55, HOCl 70, HOCl 110), BLM or PBS was subcutaneously injected in BALB/c mice daily for 6 weeks. **(A)** Skin swollenness, thickening, subcutaneous fat loss were found in the skin of HOCl and BLM group by HE and Masson staining. Lung structure was disordered, in which abundant inflammatory cells infiltrated, collagen bundles deposited in alveolar or blood vessels, and microvessels reduced in the lung of HOCl group. **(B)** Significant increase in dermal thickness was observed in HOCl and BLM group compared with those injected with PBS. **(C,D)** Skin and lung hydroxyproline content was increased in HOCl and BLM group (^*^P < 0.05).

**Figure 2:**
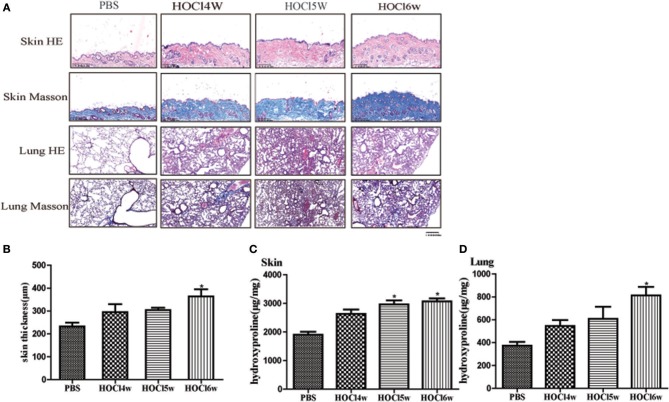
HOCl-induced dermal inflammation and fibrosis in a time-dependent manner. Mice were subcutaneously injected HOCl 110 for 4, 5, or 6 weeks. **(A)** Significant increase of inflammatory cell infiltration was found at 4 weeks and reached the peak at 5 weeks. Collagen deposition was found at 4 weeks and became obvious at 6 weeks. **(B)** An increasing dermal thickness was observed with time increasing. **(C,D)** The hydroxyproline content was also increased in the skin and lung tissues after 4 weeks (^*^P < 0.05).

We used different concentrations of HOCl to observe its effects on fibrosis. To compare the differences between HOCl and BLM, we used subcutaneous injection and intratracheal instillation of BLM, respectively, to induce mouse skin or lung fibrosis. Dermal thickness was measured on skin sections in the injection areas of BALB/c mice. Among the different HOCl concentration (HOCl55, HOCl70, and HOCl110) groups, all the HOCl groups showed skin and lung fibrosis compared with the PBS control group, as well as skin swelling, thickening, and subcutaneous fat loss. Lung structure was disordered, abundant inflammatory cells were infiltrated, collagen bundles were deposited in the alveoli or blood vessels, and microvessels were reduced in all HOCl groups. Skin and lung fibrosis were most obvious in the HOCl55 group; however, this group also had the highest mortality, while no mortality or weight loss was found in the HOCl110 group. Therefore, we selected the HOCl110 group for further analysis (Figures 1A,B). Among the different time courses of the HOCl110 groups (4, 5, and 6 weeks), a significant increase in dermal thickness was observed, up to ~50% thicker in the 6 weeks group compared with PBS group. Collagen deposition emerged from 4 weeks and gradually increased through 6 weeks. Abundant inflammatory cell infiltration was found at 4 weeks and reached the peak at 5 weeks (Figures 2A,B). Likewise, skin and lung hydroxyproline content, a marker of collagen deposition, was significantly increased in the model group compared to the control group (Figures 1C, 2C). Compared with the HOCl group, fibrosis in the skin and lung tissues of the BLM group was limited and variable. HOCl induced diffuse inflammation, fibrosis, and vasculopathy in the skin and lung of mice.

### Important Fibrosis Pathways in HOCl-Induced Mice

**Figure 3:**
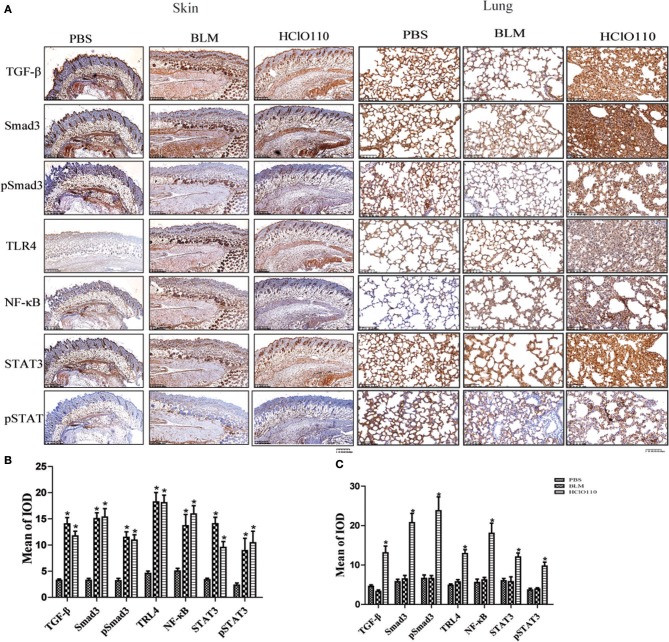
The expression of important fibrosis pathways mediators in the skin and lung tissues of HOCl induced mice. **(A)** TGF-β, NF-κB, Smad3, phospho-Smad3, STAT3, and phospho-STAT3 were strongly increased in the skin of HOCl and BLM groups, as well as the lung tissues of HOCl group. **(B,C)**The statistical analysis of the protein expression of important fibrosis mediators in skin and lung tissues (^*^P < 0.05, MOD: mean optical intensity).

To compare the differences between the BLM and HOCl-induced mouse model, BLM was intradermally injected in the following studies. TGF-β/Smad, TLR4, and IL-6/STAT3 signaling are critical pathways in SSc fibrosis. Therefore, we examined the expression of important proteins of these pathways. Significantly increased expression of TGF-β, Smad3, TLR4, NF-κB, and STAT3 were found in the fibrotic skin tissues of the BLM and HOCl-induced model (Figures 3A,B). However, in the lung tissues, high levels of these important mediators were increased in the HOCl group, but no significant alterations were found in the intradermal injection BLM group (Figures 3A,B).

### Inflammatory Infiltrates in the Skin and Lung Fibrosis

**Figure 4:**
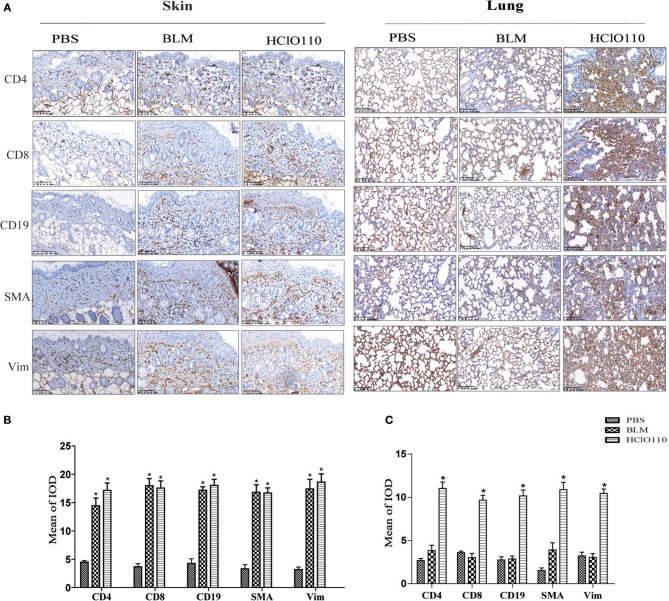
Inflammatory infiltrates in the skin and lung tissues of HOCl-induced mice. T cell (CD4+/CD8+), B cell (CD19+) and myofibroblast (SMA+/Vimentin+) were detected by immunohistochemical analysis. **(A)** The expression intensity and range of CD4 and CD8 was increased in the skin of the HOCl group and BLM group. High levels of SMA and Vim were also found in the two groups. **(B,C)** The MOD in HOCl and BLM group was significantly higher than PBS group. (^*^P < 0.05, MOD: mean optical intensity).

Previous studies revealed that T cells, B cells and fibroblasts played major roles in SSc immunological disruption. Here, we detected the types of infiltrated cells in the skin and lung tissues by immunohistochemistry. In the skin tissues, a striking increase of CD4^+^ T cells, CD8^+^ T cells, and CD19^+^ B cells was found in BLM and HOCl-induced mice, predominantly in the deep dermal layer. The expression of α-SMA and vimentin was also increased in both models, which are the markers of myofibroblasts, which indicated an increased number of myofibroblasts (Figure 4). In the lung tissues, abundant inflammatory infiltrates (CD4^+^ T cells, CD8^+^ T cells, and CD19^+^ B cells) and extensive consolidation of the lung parenchyma with alveolar architecture loss were found in HOCl-induced mice. However, no obvious changes were found in the intradermal injection BLM group (Figure 4). These results suggested that immune cells might interact with myofibroblasts and participate in the fibrosis process of the HOCl-induced mouse model.

**Figure 5:**
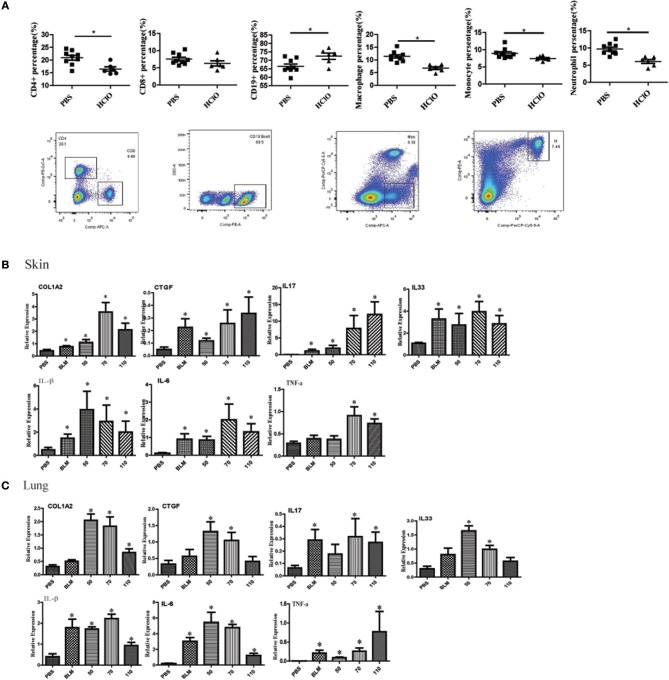
The alteration of immune cell in the spleen and pro-inflammatory mediators in the skin and lung tissues of HOCl-induced mice. **(A)** The percentage of CD4+ T cells, CD8+ T cells, CD19+ B cells, macrophages, monocytes and neutrophils in the spleen of HOCl-induced mice. **(B)** The levels of IL-1β, IL-6, IL-17, IL-33, TNF-α, CTGF, and COL1A2 mRNA in the skin and **(C)** lung tissues. All experiments were repeated at least three times. Data are presented as 2 ^(−ΔΔCT)^ relative to the levels of GAPDH. ^*^ indicates *P* < 0.05 vs. PBS group.

We also detected the proportion of immune cells in the spleen of mice. The splenic CD4+ T cells, CD8+ T cells, macrophages, monocytes, and neutrophils subpopulations were decreased in the HOCl induced fibrosis mouse, whereas the percentage of CD19^+^ B cells was increased (Figure 5A).

### The Expression of Pro-inflammatory Mediators in HOCl-Induced Mice

Many pro-inflammatory mediators contribute to inflammation and fibrosis in SSc. To explore local inflammation in skin and lung tissues of HOCl-induced mice, we examined the transcript levels of pro-inflammatory mediators such as IL-1β, IL-6, IL-17, IL-33, TNF-α, and CTGF. Among these cytokines, IL-1β, IL-6, IL-17, IL-33, and TNF-α were increased in HOCl-induced mice. Some of them were also increased in the BLM group (Figures 5B,C).

## Discussion

Animal models are critical for disease pathogenesis research and drug exploration. Currently, fibrosis animal models could not fully reflect disease progression of SSc patients. BLM-induced mice is the most widely used fibrosis model in SSc. However, many limitations were found in this model. First, subcutaneous injection of BLM causes dermal fibrosis but rarely causes lung changes in mice. Second, intratracheal instillation of BLM results in high mortality, and the lung lesions are heterogeneous and variable. Third, no autoantibodies were found in BLM-induced mice. In agreement with previous reports, in HOCl-induced mice, we confirmed this finding based on our observation of the pathological changes in the skin and lung tissues (19, 21, 25). Further, we analyzed the influence of the concentrations of HOCl and time course on inflammatory infiltrates and fibrosis in mice. Compared with the skin and lung changes induced by intradermal injections or intratracheal administration of BLM, HOCl-induced mice displayed diffuse skin and lung fibrosis, vasculopathy and inflammatory infiltrates, which may better mimic SSc pathogenesis. Therefore, this model could be helpful to understand the underlying mechanisms of SSc and search for new therapeutic targets.

The pathogenesis in HOCl-induced mice is based on the oxidative stress theory. In 1993, oxidative stress was proposed as the etiology of SSc (26). Many subsequent studies confirmed this hypothesis. High levels of oxidative stress markers and decreased antioxidant components were found in SSc (27). Some of them were correlated with disease duration, modified Rodnan skin score (mRSS), cardiovascular events, renal vascular damage, the severity of pulmonary fibrosis and immunological abnormalities (28–35). The important role of oxidative stress was also confirmed in other experimental SSc mouse models. The expression levels of protective antioxidants were reduced in the skin of the tight-skin (TSK-1/+) mouse fibrosis model (36). Overexpressed hydroxyl radicals and superoxide were found in BLM-induced mice (37). In the HOCl-induced model, subcutaneous injection of HOCl could directly induce significant inflammation, vasculopathy and fibrosis, which provides strong evidence for the critical role of oxidative stress in fibroblast activation. Therefore, further studies to investigate restoring redox homeostasis could pave the way for novel antifibrotic therapies.

In this study, we found infiltrated CD4+T cells, CD8+T cells and CD19+B cells were increased in the skin and lung tissues of the HOCl-induced mouse model. The splenic CD19^+^ B cells were increased; however, the splenic CD4+ T cells, CD8+ T cells, and macrophages, monocytes and neutrophils count were decreased. In early skin lesions, inflammatory infiltrates with lymphocytes and macrophages in perivascularly and the lower dermis and subcutis is one of the main abnormalities of SSc. Meanwhile, inflammatory infiltrates of lymphocytes, eosinophils, and macrophages in the alveolar walls is also found in early SSc (38, 39). Overexpressed autoantibodies and B cell-derived proinflammatory/profibrotic cytokines suggested that B cells were hyperactivated and played important roles in the pathogenesis of fibrosis (6, 40). Increased T cell-derived cytokines in the serum and T cell activation markers in dermal tissues also indicated T cells are activated in SSc (8, 41). In the HOCl-induced fibrosis mouse model, a similar immune cell population was found in the skin and lung tissues. However, we have not detected the subpopulations of CD4+T cells, CD8+ T cells and CD19^+^ B cell, which could provide more information on the reason of their decreased levels in the spleen.

Overall, HOCl-induced mice displayed significant tissue inflammatory infiltrates, loss of microvessels, fibrosis, high levels of pro-inflammatory mediators and important active fibrosis pathways, which closely resembled the three typical characteristics in human SSc. Here, for the first time, we evaluate the relationships between immune, inflammatory and fibrosis in this model. Based on the important role of oxidative stress in the pathogenesis of SSc, this model is better than the BLM-induced mouse model.

## Data Availability

All datasets generated for this study are included in the manuscript and/or the Supplementary Files.

## Ethics Statement

This study was carried out in accordance with the recommendations of Ethics Committee of Xiangya Hospital of Central South University. The protocol was approved by the Ethics Committee of Xiangya Hospital of Central South University.

## Author Contributions

MM participated in the study design, performed data analysis, and drafted the manuscript. JT carried out the HE and immunohistochemistry assays, Masson's trichrome staining and real-time PCR. WC, QD, BX, and NW participated in animal model construction. HZ and KW conceived the study, participated in the study design, and revised and finalized the manuscript. All authors read and approved the final manuscript.

### Conflict of Interest Statement

The authors declare that the research was conducted in the absence of any commercial or financial relationships that could be construed as a potential conflict of interest.

## References

[1] Denton CP Khanna D. Systemic sclerosis. Lancet. (2017) 390:1685–99. 10.1016/S0140-6736(17)30933-9 28413064

[2] Allanore Y Simms R Distler O Trojanowska M Pope J Denton CP Systemic sclerosis. Nat Rev Dis Primers. (2015) 1:15002 10.1038/nrdp.2015.53 27189141

[3] Bhattacharyya S Wei J Varga J. Understanding fibrosis in systemic sclerosis: shifting paradigms, emerging opportunities. Nat Rev Rheumatol. (2011) 8:42–54. 10.1038/nrrheum.2011.149 22025123 PMC3954787

[4] Zhou B Zuo XX Li YS Gao SM Dai XD Zhu HL. Integration of microRNA and mRNA expression profiles in the skin of systemic sclerosis patients. Sci Rep. (2017) 7:42899. 10.1038/srep42899 28211533 PMC5314349

[5] Zuo X Zhang L Luo H Li Y Zhu H. Systematic approach to understanding the pathogenesis of systemic sclerosis. Clin Genet. (2017) 92:365–71. 10.1111/cge.12946 27918067

[6] Forestier A Guerrier T Jouvray M Giovannelli J Lefèvre G Sobanski V. Altered B lymphocyte homeostasis and functions in systemic sclerosis. Autoimmun Rev. (2018) 17:244–255. 10.1016/j.autrev.2017.10.015 29343447

[7] Liu M Wu W Sun X Yang J Xu J Fu W. New insights into CD4(+) T cell abnormalities in systemic sclerosis. Cytokine Growth Factor Rev. (2016) 28:31–6. 10.1016/j.cytogfr.2015.12.002 26724976

[8] Slobodin G Rimar D. Regulatory T Cells in Systemic Sclerosis: a Comprehensive Review. Clin Rev Allergy Immunol. (2017) 52:194–201. 10.1007/s12016-016-8563-6 27318947

[9] Ntelis K Solomou EE Sakkas L Liossis SN Daoussis D. The role of platelets in autoimmunity, vasculopathy, and fibrosis: implications for systemic sclerosis. Semin Arthritis Rheum. (2017) 47:409–17. 10.1016/j.semarthrit.2017.05.004 28602360

[10] Mostmans Y Cutolo M Giddelo C Decuman S Melsens K Declercq H. The role of endothelial cells in the vasculopathy of systemic sclerosis: a systematic review. Autoimmun Rev. (2017) 16:774–786. 10.1016/j.autrev.2017.05.024 28572048

[11] Gilbane AJ Denton CP Holmes AM. Scleroderma pathogenesis: a pivotal role for fibroblasts as effector cells. Arthritis Res Ther. (2013) 15:215. 10.1186/ar4230 23796020 PMC4060542

[12] Biernacka A Dobaczewski M Frangogiannis N. TGF-beta signaling in fibrosis. Growth Factors. (2011) 29:196–202. 10.3109/08977194.2011.595714 21740331 PMC4408550

[13] Sgonc R Wick G. Pro- and anti-fibrotic effects of TGF-beta in scleroderma. Rheumatology. (2008) 47(Suppl. 5):v5–7. 10.1093/rheumatology/ken275 18784145 PMC3292795

[14] Zhao J Shi W Wang YL Chen H Bringas P Datto MB. Smad3 deficiency attenuates bleomycin-induced pulmonary fibrosis in mice. Am J Physiol Lung Cell Mol Physiol. (2002) 282:L585–93. 10.1152/ajplung.00151.2001 11839555

[15] Warburton D Shi W Xu B. TGF-beta-Smad3 signaling in emphysema and pulmonary fibrosis: an epigenetic aberration of normal development? Am J Physiol Lung Cell Mol Physiol. (2013) 304:L83–5. 10.1152/ajplung.00258.2012 23161884 PMC4073936

[16] Seki E De Minicis S Osterreicher CH Kluwe J Osawa Y Brenner DA. TLR4 enhances TGF-beta signaling and hepatic fibrosis. Nat Med. (2007) 13:1324–32. 10.1038/nm1663 17952090

[17] Marangoni RG Varga J Tourtellotte WG. Animal models of scleroderma: recent progress. Curr Opin Rheumatol. (2016) 28:561–70. 10.1097/BOR.0000000000000331 27533324

[18] Del Galdo F Matucci-Cerinic M. The search for the perfect animal model discloses the importance of biological targets for the treatment of systemic sclerosis. Ann Rheum Dis. (2014) 73:635–6. 10.1136/annrheumdis-2013-203910 24257026

[19] Servettaz A Goulvestre C Kavian N Nicco C Guilpain P Chéreau C. Selective oxidation of DNA topoisomerase 1 induces systemic sclerosis in the mouse. J Immunol. (2009) 182:5855–64. 10.4049/jimmunol.0803705 19380834

[20] Morin F Kavian N Nicco C Cerles O Chéreau C Batteux F. Niclosamide prevents systemic sclerosis in a reactive oxygen species-induced mouse model. J Immunol. (2016) 197:3018–28. 10.4049/jimmunol.1502482 27613696

[21] Morin F Kavian N Chouzenoux S Cerles O Nicco C Chéreau C. Leflunomide prevents ROS-induced systemic fibrosis in mice. Free Radic Biol Med. (2017) 108:192–203. 10.1016/j.freeradbiomed.2017.03.035 28365359

[22] Zhu H Luo H Li Y Zhou Y Jiang Y Chai J. MicroRNA-21 in scleroderma fibrosis and its function in TGF-beta-regulated fibrosis-related genes expression. J Clin Immunol. (2013) 33:1100–9. 10.1007/s10875-013-9896-z 23657402

[23] Liang M Lv J Zou L Yang W Xiong Y Chen X. A modified murine model of systemic sclerosis: bleomycin given by pump infusion induced skin and pulmonary inflammation and fibrosis. Lab Invest. (2015) 95:342–50. 10.1038/labinvest.2014.145 25502178

[24] Zhang L Zhu H Li Y Dai X Zhou B Li Q. The role of IFI35 in lupus nephritis and related mechanisms. Mod Rheumatol. (2017) 27:1010–8. 10.1080/14397595.2016.1270387 28064541

[25] Kavian N Marut W Servettaz A Nicco C Chéreau C Lemaréchal H. Reactive oxygen species-mediated killing of activated fibroblasts by arsenic trioxide ameliorates fibrosis in a murine model of systemic sclerosis. Arthritis Rheum. (2012) 64:3430–40. 10.1002/art.34534 22576901

[26] Yancey KB A radical proposal for the pathogenesis of scleroderma. J Am Acad Dermatol. (1993) 28:78–85. 8425975 10.1016/0190-9622(93)70014-k

[27] Luo JY Liu X Jiang M Zhao HP Zhao JJ. Oxidative stress markers in blood in systemic sclerosis: a meta-analysis. Mod Rheumatol. (2017) 27:306–14. 10.1080/14397595.2016.1206510 27425641

[28] Tikly M Channa K Theodorou P Gulumian M. Lipid peroxidation and trace elements in systemic sclerosis. Clin Rheumatol. (2006) 25:320–4. 10.1007/s10067-005-0013-4 16249831

[29] Riccieri V Spadaro A Fuksa L Firuzi O Saso L Valesini G. Specific oxidative stress parameters differently correlate with nailfold capillaroscopy changes and organ involvement in systemic sclerosis. Clin Rheumatol. (2008) 27:225–30. 10.1007/s10067-007-0769-9 17965907

[30] Böger RH Maas R Schulze F Schwedhelm E. Elevated levels of asymmetric dimethylarginine (ADMA) as a marker of cardiovascular disease and mortality. Clin Chem Lab Med. (2005) 43:1124–9. 10.1515/CCLM.2005.196 16197309

[31] Ogawa F Shimizu K Muroi E Hara T Hasegawa M Takehara K. Serum levels of 8-isoprostane, a marker of oxidative stress, are elevated in patients with systemic sclerosis. Rheumatology. (2006) 45:815–8. 10.1093/rheumatology/kel012 16449367

[32] Servettaz A Guilpain P Goulvestre C Chéreau C Hercend C Nicco C. Radical oxygen species production induced by advanced oxidation protein products predicts clinical evolution and response to treatment in systemic sclerosis. Ann Rheum Dis. (2007) 66:1202–9. 10.1136/ard.2006.067504 17363403 PMC1955145

[33] Aden N Shiwen X Aden D Black C Nuttall A Denton CP. Proteomic analysis of scleroderma lesional skin reveals activated wound healing phenotype of epidermal cell layer. Rheumatology. (2008) 47:1754–60. 10.1093/rheumatology/ken370 18829709

[34] Murray AK Moore TL Manning JB Griffiths CE Herrick AL. Noninvasive measurement of skin autofluorescence is increased in patients with systemic sclerosis: an indicator of increased advanced glycation endproducts? J Rheumatol. (2012) 39:1654–8. 10.3899/jrheum.111359 22753661

[35] Avouac J Borderie D Ekindjian OG Kahan A Allanore Y. High DNA oxidative damage in systemic sclerosis. J Rheumatol. (2010) 37:2540–7. 10.3899/jrheum.100398 20843906

[36] Dooley A Low SY Holmes A Kidane AG Abraham DJ Black CM. Nitric oxide synthase expression and activity in the tight-skin mouse model of fibrosis. Rheumatology. (2008) 47:272–80. 10.1093/rheumatology/kem303 18238792

[37] Oberley LW Buettner GR. The production of hydroxyl radical by bleomycin and iron (ii). FEBS Lett. (1979) 97:47–9. 10.1016/0014-5793(79)80049-6 24754079

[38] Katsumoto TR Whitfield ML Connolly M. The pathogenesis of systemic sclerosis. Annu Rev Pathol. (2011) 6:509–37. 10.1146/annurev-pathol-011110-130312 21090968

[39] Laurent P Sisirak V Lazaro E Richez C Duffau P Blanco P. Innate immunity in systemic sclerosis fibrosis: recent advances. Front Immunol. (2018) 9:1702. 10.3389/fimmu.2018.01702 30083163 PMC6064727

[40] Sakkas LI Bogdanos DP. Systemic sclerosis: new evidence re-enforces the role of B cells. Autoimmun Rev. (2016) 15:155–61. 10.1016/j.autrev.2015.10.005 26497107

[41] Fuschiotti P. Current perspectives on the role of CD8+ T cells in systemic sclerosis. Immunol Lett. (2018) 195:55–60. 10.1016/j.imlet.2017.10.002 28987475 PMC5820151

