# Supplementary material for: The Fibrosis and Immunological Features of Hypochlorous Acid Induced Mouse Model of Systemic Sclerosis
Source: Front Immunol. 2019 Aug 20;10:1861. doi: 10.3389/fimmu.2019.01861 (PMC6710365; doi:10.3389/fimmu.2019.01861)
Supplement: Supplementary file 1 [file Data_Sheet_1.pdf]

Table S1. Real-Time PCR primer sequence

Primer	Primer Sequence (5'to3')
COL1A2-F	TTCTGTGGGTCCTGCTGGGAAA
COL1A2-R	TTGTCACCTCGGATGCCTTGAG
IL-6-F	TACCACTTCACAAGTCGGAGGC
IL-6-R	CTGCAAGTGCATCATCGTTGTTC
IL-17-F	CAGACTACCTCAACCGTTCCAC
IL-17-R	TCCAGCTTTCCCTCCGCATTGA
IL-33-F	CTACTGCATGAGACTCCGTTCTG
IL-33-R	AGAATCCCGTGGATAGGCAGAG
CTGF-F	TGCGAAGCTGACCTGGAGGAAA
CTGF-R	CCGCAGAACTTAGCCCTGTATG
IL-β-F	TGGACCTTCCAGGATGAGGACA
IL-β-R	GTTCATCTCGGAGCCTGTAGTG
TNF-F	GGTGCCTATGTCTCAGCCTCTT
TNF-R	GCCATAGAACTGATGAGAGGGAG

F: Forward; R: Reverse
